# Ferroelectric-tuned van der Waals heterojunction with band alignment evolution

**DOI:** 10.1038/s41467-021-24296-1

**Published:** 2021-06-29

**Authors:** Yan Chen, Xudong Wang, Le Huang, Xiaoting Wang, Wei Jiang, Zhen Wang, Peng Wang, Binmin Wu, Tie Lin, Hong Shen, Zhongming Wei, Weida Hu, Xiangjian Meng, Junhao Chu, Jianlu Wang

**Affiliations:** 1 State Key Laboratory of Infrared Physics, Shanghai Institute of Technical Physics, Chinese Academy of Sciences, Shanghai, China; 2 School of Physics and Electronic Science, East China Normal University, Shanghai, China; 3 State Key Laboratory of Superlattices and Microstructures, Institute of Semiconductors, Chinese Academy of Sciences, Beijing, China; 4 Hangzhou Institute for Advanced Study, University of Chinese Academy of Sciences, Chinese Academy of Sciences, Hangzhou, China

**Keywords:** Electronic devices, Nanophotonics and plasmonics

## Abstract

Van der Waals integration with abundant two-dimensional materials provides a broad basis for assembling functional devices. In a specific van der Waals heterojunction, the band alignment engineering is crucial and feasible to realize high performance and multifunctionality. Here, we design a ferroelectric-tuned van der Waals heterojunction device structure by integrating a GeSe/MoS_2_ VHJ and poly (vinylidene fluoride-trifluoroethylene)-based ferroelectric polymer. An ultrahigh electric field derived from the ferroelectric polarization can effectively modulate the band alignment of the GeSe/MoS_2_ heterojunction. Band alignment transition of the heterojunction from type II to type I is demonstrated. The combination of anisotropic GeSe with MoS_2_ realizes a high-performance polarization-sensitive photodetector exhibiting low dark current of approximately 1.5 pA, quick response of 14 μs, and high detectivity of 4.7 × 10^12^ Jones. Dichroism ratios are also enhanced by ferroelectric polarization in a broad spectrum from visible to near-infrared. The ferroelectric-tuned GeSe/MoS_2_ van der Waals heterojunction has great potential for multifunctional detection applications in sophisticated light information sensing. More profoundly, the ferroelectric-tuned van der Waals heterojunction structure provides a valid band-engineering approach to creating versatile devices.

## Abstract (web summary)

Band alignment engineering is important to realize high performance and multifunctionality in a specific van der Waals heterojunction. Here, the authors observe band alignment transition of the heterojunction in a ferroelectric-tuned van der Waals heterojunction device with high performance.

## Introduction

Heterojunctions are an essential part of functional devices. The integration of different materials guarantees the acquisition of high-performance heterojunctions. Traditional integration strategies, such as chemical and physical epitaxial growth, commonly require harsh preparation conditions and rarely achieve the ideal interface. Interface disorder, chemical contamination, strain, and diffusion effects limit the performance and application of heterostructures^1–3^. Emerging two-dimensional (2D) layered materials provide a state-of-the-art approach to designing heterojunctions based on van der Waals (vdW) interaction. A vdW heterojunction (VHJ) is bond-free integration that is unaffected by the lattice mismatch or the processing condition of materials used. As basic building blocks, various 2D materials exhibit impressive electrical, optical, and magnetic properties^4–6^. Moreover, vdW integration with an atomically clean surface in the heterostructure is notably superior to those of only one material^7,8^. An excellent example is the twisted integration of two monolayer graphene that exhibit unconventional superconductivity and correlated insulator behavior^9,10^. In addition to fundamental physics research, studies have reported that VHJs have excellent potential in high-performance multifunctional devices. Optoelectronic devices, such as photovoltaic photodetectors^11,12^ and quantum well LEDs^13^, have attracted tremendous research interest because they exhibit remarkably better performance than traditional devices. Electronic devices such as floating-gate memories^14,15^ and tunneling diodes^16–20^ have exhibited great potential for future applications as well.

With band structure design, the richness of 2D materials and the flexibility of vdW integration provide numerous routes to realize specific functional devices. Typically, three different band alignments can be formed in VHJs: straddling (type I), staggered (type II), and broken (type III). However, multifunctional applications are challenging to achieve with a fixed band alignment. Reliable approaches, like applying an external field^19,21^ or controlling the doped element concentration^20,22^ band alignment change from type II to type III, can be realized. Thus, Esaki diode and rectifier diode are achieved in VHJs. Woo Jong Yu et al. used an external gate to modulate the band slope and photocurrent generation of graphene-MoS_2_-graphene vertical heterostructure with high quantum efficiency^12^. By sufficient controlling of an external electric field, VHJs can achieve robust performance and expand diverse application potentials.

However, tuning of the Fermi levels by the electric field is insufficient to change the band offset, which affects the performance of a heterojunction significantly. Besides, a sustained external voltage to maintain the optimal performance of the device causes considerable power consumption. Ferroelectrics provide a non-volatile remanent polarization electric field over 1 V/nm^23–25^, which is large enough to modify the bandgap of semiconductors according to the Stark effect^26–29^. Ferroelectric field-effect transistors based on ferroelectrics and 2D materials have been demonstrated high performances and great potentials in electronics and optoelectronics^30–32^. Predictably, combining ferroelectrics with VHJs achieves highly tunable band structures and tailored optoelectronic properties. Here, we designed a ferroelectric VHJ (Fe-VHJ) consisting of a ferroelectric polymer, P(VDF-TrFE), and GeSe/MoS_2_ VHJ. Under modulation of the ferroelectric polarization field, the electrical characteristics of the GeSe/MoS_2_ *pn* junction can be tuned and its output can be maintained at two specific states. The band alignment between GeSe and MoS_2_ can be switched from type II (staggered) to type I (straddling) by reversing the direction of ferroelectric polarization field. With ferroelectric-tuned VHJ band engineering, the GeSe/MoS_2_ junction can act as a high-performance photodetector. High responsivities are achieved for a wide range of wavelengths spanning from visible to near-infrared. The anisotropy of GeSe is employed to demonstrate a polarized light-sensitive photodetector, which is further enhanced by the ferroelectric field. More importantly, the ferroelectric field is applied on the VHJ to engineer the band alignment type, broaden the detection wavelength range, and enrich functionalities.

## Results and discussion

### Fabrication of GeSe/MoS_2_ Fe-VHJ

**Fig. 1:**
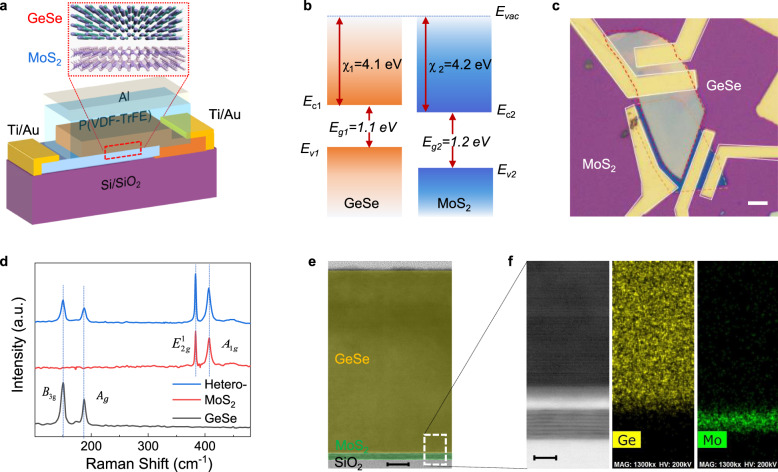
Design and characterization of GeSe/MoS_2_ heterojunction. **a** Schematic illustration of the GeSe/MoS_2_ heterojunction with the P(VDF-TrFE) gate. **b** Energy bands of GeSe and MoS_2_ before contact. *χ*_1_, *χ*_2_, *E*_g1_, and *E*_g1_ are from related literatures. **c** Optical micrograph of the heterojunction; scale bar: 5 μm. **d** Raman spectra of GeSe and MoS_2_ and their overlap. **e** TEM image of the heterostructure; scale bar: 10 nm. **f** High-resolution cross-sectional image of the GeSe/MoS_2_ interface and the corresponding chemical element distribution; scale bar: 5 nm.

The device structure we designed is depicted in Fig. 1a. GeSe and MoS_2_ form the VHJ, in which GeSe is an anisotropic 2D semiconductor. Unlike MoS_2_, which has an in-plane hexagonal structure, GeSe has a rectangular structure in the plane, that is, different structures in the *x* and *y* directions. It has an “armchair” shape along the *x*-axis and a “zigzag” shape along the *y*-axis. The specific atomic structure of GeSe is illustrated in Supplementary Fig. 1. Because of its anisotropic structure, GeSe possesses electrical anisotropy and optical linear dichroism, which satisfies its application in polarized light detection. P(VDF-TrFE) is covered on the GeSe/MoS_2_ VHJ. Here we apply it to engineer the band structure and enhance the optoelectronic performance of the VHJ. The band structure of GeSe and MoS_2_ is shown in Fig. 1b. The bottom of the conduction band and the top of the valence band of GeSe (MoS_2_) are approximately −4.1 eV (−4.2 eV) and −5.2 eV (−5.4 eV)^33^. The offsets of the conduction band and valence band are approximately 0.1 and 0.2 eV, respectively. Therefore, a type II band alignment is formed, which is beneficial for separating photo-generated carriers and achieving high-speed photoresponse. Figure 1c is an optical micrograph of the device. Electrodes are situated near the boundary of the overlapped heterojunction to eliminate the effect of series resistance. The heterostructure composed of GeSe and MoS_2_ is well assembled, as confirmed by Raman spectra and transmission electron microscopy (TEM). As shown in Fig. 1d, the heterostructure exhibits Raman characteristic peaks of both GeSe and MoS_2_. The peaks at 150.6 and 187.4 cm^−1^ represent the out-of-plane vibration mode (*B*_3g_) and in-plane vibration mode (*A*_g_) of GeSe, respectively. The other two peaks at 383.5 cm^−1^ (in-plane vibration mode *E*^1^_2g_) and 407.5 cm^−1^ (out-of-plane vibration mode *A*_1g_) belong to MoS_2_. The cross-section of the heterojunction observed by TEM is displayed in Fig. 1e. The thickness of GeSe is approximately 120 nm, and that of MoS_2_ is approximately 6 nm. The GeSe nanoflake is relatively thick for better light absorption, and the thin MoS_2_ is chosen for the effective collection of photo-generated electrons. A similar design has been reported in BP/MoS_2_ photodetectors^34^. The vdW integration of GeSe and MoS_2_ does not exhibit mismatch or pinning on the interface, thus guaranteeing high electrical performance. In this device, a thin amorphous layer is observed in the high-resolution image of the GeSe/MoS_2_ interface (Fig. 1f). This layer possibly originates from the exfoliation-restack process, which may hinder the separation of photo-generated carriers and delay the response time^34^. The corresponding feature element maps obtained through energy-dispersive X-ray spectroscopy depict the spatial distribution of GeSe and MoS_2_.

### Electrical properties and operation mechanism of the device

**Fig. 2:**
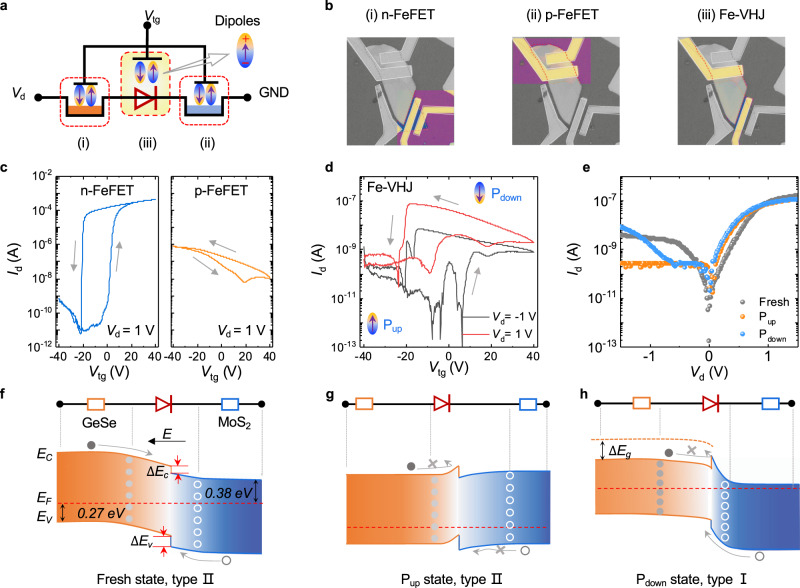
Electrical properties of GeSe/MoS_2_ heterojunction tuned by the ferroelectric polymer P(VDF-TrFE). **a** Electrical configuration of the device, with three parts represented in the dashed-line frames. **b** Optical image of three device elements: MoS_2_ n-FeFET, GeSe p-FeFET, and GeSe/MoS_2_ Fe-VHJ. **c** Transfer characteristics of GeSe p-FeFET and MoS_2_ n-FeFET at *V*_d_ = 1 V. **d** Transfer characteristics of the Fe-VHJ under forward and reverse biases. **e** Output characteristics of the GeSe/MoS_2_ heterojunction when P(VDF-TrFE) are fresh, polarized upward, and polarized downward states. **f** Schematic of the energy band structure of the GeSe/MoS_2_ junction at “Fresh” state. *E*_F_-*E*_C_ and *E*_F_-*E*_v_ were calculated using experimental data. Schematic of the heterojunction energy bands at an equilibrium state. Bandgap evolution of the GeSe/MoS_2_ Fe-VHJ at **g** “P_up_” state and **h** “P_down_” state.

As depicted in Fig. 2a, the device configuration consists of three functional parts in a series-resistance mode: a p-type GeSe ferroelectric field-effect transistor (p-FeFET), an n-type MoS_2_ ferroelectric field-effect transistor (n-FeFET), and a GeSe/MoS_2_ Fe-VHJ. We can decide which one is dominant by changing the gate or bias voltage. Figure 2b presents a micrograph of the device; the highlighted portion represents the actual test area, corresponding to the three parts in Fig. 2a. From the output curves in Supplementary Fig. 2, both MoS_2_ and GeSe are well-contacted with their electrodes, indicating that the Schottky barrier has little effect on the electrical performance of the VHJ. The transfer characteristics of n-type MoS_2_ and p-type GeSe gated by P(VDF-TrFE) are portrayed in Fig. 2c, which shows the same hysteresis direction. The transfer curve of MoS_2_ exhibits ferroelectric hysteresis, whereas that of GeSe does not. The counter-clockwise hysteresis of GeSe originates from charge injection or carriers capture by trap sites. Due to the interface trap states, a large hysteresis window is found in the transfer curve of a GeSe FET tuned by SiO_2_ back gate (Supplementary Fig. 3b). By contrast, under the modulation of P(VDF-TrFE) top gate, part of the trap states is localized by the polarization field, thereby narrowing the hysteresis window (Supplementary Fig. 3a). Furthermore, two peaks in gate current are generated by polarization state reverse. So, the polarization electric field is indeed applied to the channel. The ferroelectric electric field (~1.2 V/nm) is giant enough to modulate the intrinsic carrier concentration (*n*_i_ ∝ exp(–*E*_g_/2*kT*)) and broaden the photoresponse spectral range as shown in Supplementary Figs. 4 and 5. Therefore, the P(VDF-TrFE) is supposed to modulate the band structure of GeSe. Detailed DFT calculations and experimental analysis are provided in Supplementary Figs. 6–8 and Supplementary Notes 1 and 2. The transfer characteristics of the VHJ gated by P(VDF-TrFE) are shown in Fig. 2d. In the device configuration, the heterojunction is in series with MoS_2_ and GeSe, so the transfer characteristics of the device are also co-modulated by MoS_2_ and GeSe nanoflakes. The ferroelectric hysteresis is observed in the transfer curves at a forward bias (1 V) and a reverse bias (−1 V). The transport plots can be sorted into two parts. When *V*_tg_ sweeps from −40 to 20 V and from −20 to −40 V, P(VDF-TrFE) polymer is polarized up (P_up_) and MoS_2_ channel is fully depleted, which turns off the entire channel and results in the smallest *I*_d_. When *V*_tg_ sweeps from 40 to −20 V and from 20 to 40 V, P(VDF-TrFE) polymer is polarized down (P_down_), the MoS_2_ channel is turned on and most *V*_d_ loads on the p-type GeSe part. As a result, holes in GeSe domain the electrical properties of the channel and the drain current increases when the gate voltage sweeps from 40 to −20 V. Therefore, the carrier concentration of the Fe-VHJ is modulated by polarization field, and the device can maintain two states without external gate voltage. Figure 2e shows the output characteristics at three states: “Fresh”, “P_up_”, and “P_down_”, corresponding to the different polarization states of P(VDF-TrFE). At the Fresh state, the GeSe/MoS_2_ junction acts as a *pn* diode, but its rectification ratio is small. The ferroelectric polarization states have considerable impacts on reverse current, whereas forward current remains almost unchanged. The changes in the reverse current may result from the band structure transform at the heterojunction interface.

The experimental determination of band structures of semiconductors and heterojunctions includes optical techniques^28,35^, photoemission spectroscopy^28,36–38^, and electrical transport techniques^39,40^. For the limitation of materials and device structure, the photoemission and optical techniques are not suitable for our device. Here, we analyze the band structure evolution with polarization electric field from the electrical transport properties and the phenomena model is built in Figs. 2f–h. At the fresh state, GeSe and MoS_2_ formed a type II heterojunction, which is conducive to photo-generated carrier separation. According to Fig. 1b, the band offsets of the conduction bands and the valence bands are 0.10 and 0.20 eV, respectively. In this GeSe/MoS_2_ VHJ, the difference between the Fermi level and the bottom of the valence band of GeSe is 0.27 eV, and the difference between the Fermi level and the top of the conduction band of MoS_2_ is 0.38 eV. They are derived from the formulas: *n* = (*g*_*2D*_*k*_*B*_*T*)*ln*{*1* + *exp*[(*E*_*F*_*-E*_*C*_)*/k*_*B*_*T*]} and *p* = (*g*_*2D*_*k*_*B*_*T*)*ln*{*1* + *exp*[−(*E*_*F*_*-E*_*V*_)*/k*_*B*_*T*]}, where *g*_*2D*_ is 2D density of states in TMD semiconductor. The carrier density *n* and *p* are calculated by *n*(*p*) = *σ*/*μe*, where conductivity *σ* and mobility *μ* are extracted from the output and transfer curves in Supplementary Fig. 2. The calculation details are displayed in Supplementary Note 3. Consequently, the difference between the Fermi levels of these two materials can be calculated to be 0.35 eV before they contact. At P_up_ state, holes in GeSe are accumulated and electrons in MoS_2_ are depleted, leading to the band bending upward in GeSe. The drift current of minority carriers in both sides is restricted, resulting in suppression of reverse current. The reverse current is approximately 0.2 nA, lower than that at the Fresh state. When P(VDF-TrFE) is polarized down, the reverse current is suppressed under small bias but increases under larger bias. The increased current is generated from Fowler–Nordheim tunneling as fitted in Supplementary Fig. 10, indicating the existence of a triangle barrier on the conduction band. The triangle barrier may result from the bandgap reduction of GeSe at P_down_ state. Generally, an electrical field narrows the bandgap of semiconductors by shifting the conduction band minimum downward while the conduction band maximum is nearly a constant^27,41^. Thus, the band structure evolution of GeSe changes the conduction band offset from 0.10 eV to a negative value thus type I (straddling) band alignment is formed. Although the band offset ∆*E*_*c*_ is a small value, it has an effective impact on carrier transport as shown in the temperature-dependent electrical properties in Supplementary Fig. 9. Hereto, based on these electrical analyses, it is verified that the band alignment of the GeSe/MoS_2_ can be effectively tuned by the ferroelectric field. According to the Debye length of GeSe, the tunability of ferroelectric field can still work on thicker GeSe as analyzed in Supplementary Note 4.

### Performance of photovoltaic response

**Fig. 3:**
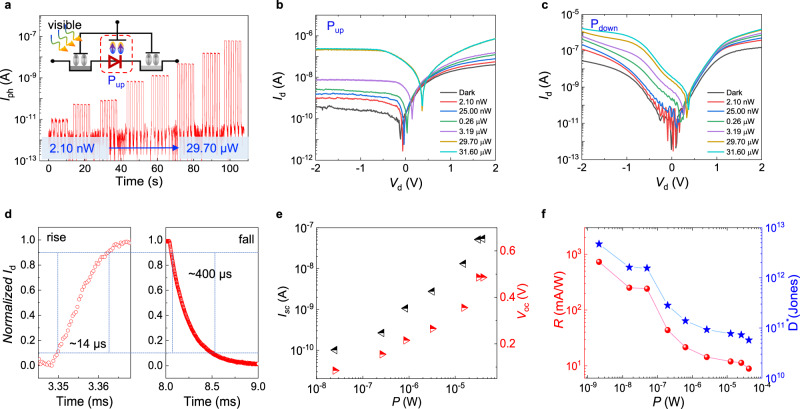
Photoresponse performance of GeSe/MoS_2_ Fe-VHJ in the visible region. **a** Time-resolved photocurrent in the “P_up_” state with 520 nm incident light and power are 2.10 nW, 25.00 nW, 50.10 nW, 0.26 μW, 0.85 μW, 3.19 μW, 15.30 μW, and 29.70 μW, respectively. The light power is the fraction on the heterojunction. Output characteristics under different light powers at **b** “P_up_” state and **c** “P_down_” state. **d** In all, 10–90% photocurrent rise and decay times derived with λ = 520 nm illumination. **e** Power dependency of short-circuit current and open-circuit voltage with an incident light wavelength of 520 nm. **f** Photoresponsivity and detectivity of GeSe/MoS_2_ Fe-VHJ.

To characterize the photodetection performance of GeSe/MoS_2_ Fe-VHJ, we tested its photoresponse from visible to near-infrared. To ensure a low and stable dark current, the P(VDF-TrFE) polymer is polarized up. As presented in Fig. 3a, the heterojunction demonstrates ultra-low dark current at *V*_d_ = 0 V and embraces highly sensitive photoresponse to 520 nm illumination with different incident light power. The *I*_d_–*V*_d_ characteristics under different illumination intensities at P_up_ and P_down_ states are shown in Fig. 3b, c, respectively. The injection of photo-generated carriers makes the *I*_d_–*V*_d_ curves shift, resulting in short-circuit current (*I*_sc_) and open-circuit voltage (*V*_oc_). The *V*_oc_ reaches 0.49 V at P_up_ state that is larger than 0.37 V of P_down_ state. The difference of *V*_oc_ indicates that the difference of Fermi levels is tuned by the electric field originated from ferroelectric polarization. Moreover, the dark current is suppressed at P_up_ state, leading to a larger photoswitching on/off ratio and improving the sensitivity. Therefore, the Fe-VHJ shows a better photovoltaic response at P_up_ state, where GeSe/MoS_2_ forms a type II band alignment. As shown in Fig. 3d, the photocurrent rise time is as short as 14 μs and the fall time is 400 μs. The trap in thick GeSe is a factor limiting the response speed because it can induce photogating effect that prolonged the photo-generated carriers. Besides, the defective interfacial layer between GeSe and MoS_2_ may make a negative effect on the response speed too. *I*_sc_ and *V*_oc_ increased with the power of 520 nm light illumination, as indicated in Fig. 3e. When the device is saturated-absorption, they reach maximum values of 52 nA and 0.49 V. Figure 3f shows the power-dependence of photoresponsivity (*R*) and detectivity (D^*^) at *V*_d_ = 0 V. Photoresponsivity is calculated using the formula *R* = *I*_ph_/*P*. In a photodiode, the thermal noise and the generation-recombination (g-r) noise can all be ignored because the carriers are depleted in the space charge region. The shot noise in the current passing through the junction is the intrinsic noise. So the detectivity of GeSe/MoS_2_ photovoltaic detector can be calculated by D^*^ = *A*^1/2^*R*/(2*eI*_dark_)^1/2^, where *A* is the effective area of the photodetector (2 × 10^−6^ cm^2^), *e* is the elementary electronic charge, and *I*_dark_ is the dark current (1.5 pA). The photodetector achieves maximum photoresponsivity and detectivity values of 729.3 mA/W and 4.7 × 10^12^ Jones under the illumination of 520 nm light with 2.1 nW. To verify the specific value of the GeSe/MoS_2_ heterojunction, we measured the noise current spectrum at room temperature, as shown in Supplementary Fig. 11. The noise is directly measured allowing D^*^ to be calculated as D^*^ = (*A∆f*)^1/2^/*NEP*, where *∆f* is the integration time (1 s) and *NEP* is noise equivalent power (defined by in *i*_*n*_*/R*, where *R* is the responsivity, and *i*_*n*_ is the measured noise current)^42^. The measured low-frequency noise equivalent power can reach a value of 1.37 × 10^−15^ WHz^−1/2^. From this measurement, we find a D^*^ value of 1.03 × 10^12^ cm Hz^1/2^ W^−1^. This value is in good agreement with the result above. The small difference can be attributed to weak generation-recombination noise generated in the thick GeSe.

### Photoresponse in NIR of GeSe/MoS_2_ Fe-VHJ

**Fig. 4:**
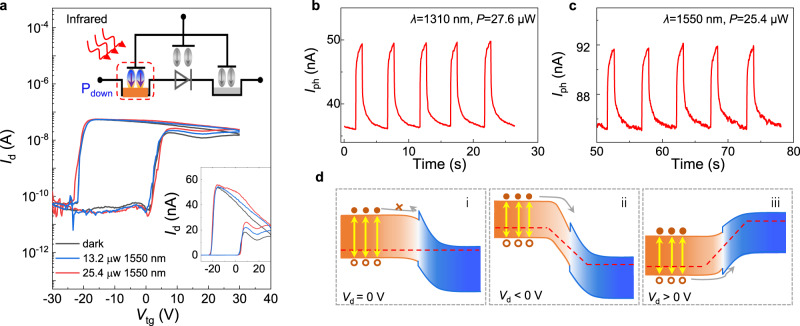
Photoresponse in near-infrared spectroscopy. **a** Transfer curves under the illumination of 1550 nm light at *V*_d_ = 1 V. Time-resolved photocurrent with incident light wavelengths of **b** 1310 nm (P_down_ state, *V*_d_ = –1 V) and **c** 1550 nm (P_down_ state, *V*_d_ = 1 V). **d** Transport of the carriers generated by infrared light in GeSe/MoS_2_ Fe-VHJ at (i) equilibrium state, (ii) reverse bias, and (iii) forward bias.

In commercial integrated photonics based on Si or Ge, transceivers, modulators, and detectors operate at a wavelength between 1300 and 1550 nm (corresponding *E*_opt_ ≈ 0.8 eV)^43–45^. According to the bandgaps of MoS_2_ (1.2 eV) and GeSe (1.1 eV), however, the application in integrated photonics of the GeSe/MoS_2_ heterojunction is spectrally limited. Nevertheless, with the Stark effect induced by the giant ferroelectric field on GeSe, the GeSe/MoS_2_ Fe-VHJ is sensitive to photons with lower energy. As demonstrated by the transfer curves at *V*_d_ = −1 V with/without 1550 nm light illumination plotted in Fig. 4a, photocurrent is generated when gate voltage sweeps from 0 V to 40 V and 40 V to −20 V, where P(VDF-TrFE) is polarized down and the GeSe/MoS_2_ heterojunction is type I alignment. A similar response is observed in transfer curves at *V*_d_ = 1 V. Photocurrent maps confirm the sensitivity of the whole GeSe area under near-infrared irradiation (Supplementary Fig. 12), while only the overlapping heterojunction part shows sensitivity to visible light. This indicates that the photocurrent is generated in the GeSe part with Stark effect works. In addition, the photocurrent switching characteristics under the illumination of 1310 and 1550 nm are shown in Fig. 4b, c, respectively. The GeSe/MoS_2_ Fe-VHJ shows stable photocurrent switching characteristics at both reverse and forward biases. In contrast to the response to visible light, the response to NIR light with assistance of an external bias is much slower (see Supplementary Fig. 13) because the device operates in a photoconductive mode in the near-infrared region. The energy band diagrams of GeSe/MoS_2_ VHJ under various biases when P(VDF-TrFE) is polarized down are shown in Fig. 4d. At equilibrium state, photo-generated carriers in GeSe cannot overcome the peak in the conduction band, falling to be collected by electrodes. With a reverse bias applied across the outer electrodes, the peak barrier is covered by the steep band structure and no longer affects the transport of electrons. Correspondingly, the band offset in the valence band is also negligible for collecting photo-generated holes when a high enough forward bias is applied. As a result, the photocurrent generated by infrared light can only be collected with a large enough bias. Therefore, band alignment evolution in GeSe/MoS_2_ FeFET broadens the detective range and makes its potential application in telecommunication based on photonic integrated circuits.

### Polarization-resolved sensitivity of GeSe/MoS_2_ Fe-VHJ

**Fig. 5:**
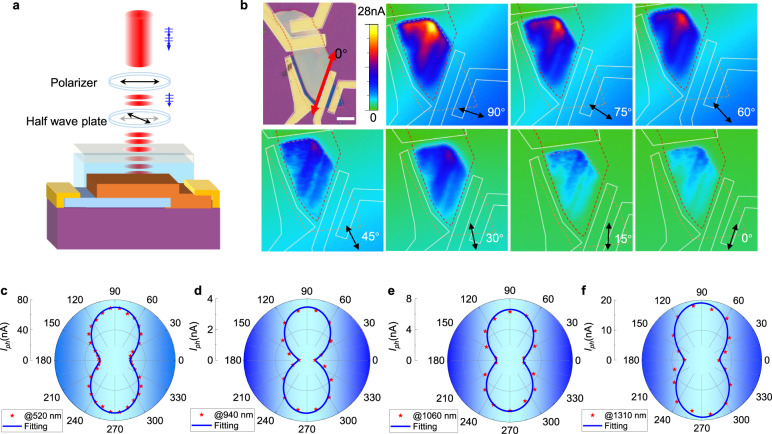
Polarization detection characteristics of the GeSe/MoS_2_ Fe-VHJ. **a** Schematic of the test setup. **b** Photocurrent mapping of the GeSe/MoS_2_ Fe-VHJ. **c**–**f** Photocurrent as a function of the incident light polarization angle. The wavelength ranges from visible to near-infrared wavelengths (520, 940, 1060, and 1310 nm). The solid line represents fitting using a sinusoidal function *I*_ph_(*δ*) = *I*_py_ cos^2^(*δ* + *φ*) + *I*_px_ sin^2^(*δ* + *φ*). The fitted dichroic ratios under different wavelengths are 5.53, 6.25, 3.80, and 3.16.

Photodetectors are essential sensing tools for acquiring light information of the environment. With the development of artificial intelligence, more accurate information must be acquired and analyzed. Anisotropic materials such as black phosphorus (BP), ReS_2_, ReSe_2_, and GeSe have been successively employed to develop polarization detectors^46–48^. Among these materials, GeSe is a promising and rare *p*-type semiconductor that can form ideal *pn* junctions with *n*-type 2D materials^33,49^. More importantly, GeSe has a direct bandgap for high-efficiency absorption^50^. As shown in Fig. 5, the polarization-dependent photoresponse of the GeSe/MoS_2_ Fe-VHJ is comprehensively studied. The test setup is illustrated in Fig. 5a. The incident light was emitted perpendicularly onto the device and polarized using a Glan–Taylor prism. By rotating the half-wave plate, the polarization angle of the linearly polarized light was altered, but the laser power remained stable. To determine the effective photoresponse area, we performed photocurrent mapping on the device. In addition, we measured the fluctuation of photocurrent to different angles of incident light. The results are presented in Fig. 5b. P(VDF-TrFE) maintains an upward polarization field during scanning, and no bias is applied to the GeSe/MoS_2_ heterojunction. Zero degree is set along with the direction of a cleavage side of GeSe. The photocurrent mappings are successively obtained from 0° to 90° by steps of 15°. The photocurrent intensity varies noticeably with the incident polarization angle. The most significant photoresponse is achieved at 90° and the weakest at 0°. According to the device configuration, the response area is the overlapping part of GeSe and MoS_2_, indicating that these two materials formed an effective *pn* junction. The specific relationship between photocurrent and polarization angle must be discovered to quantitatively characterize the polarization sensitivity of the device. Figure 5c–f shows the relationship between the photocurrent and polarization angle of the device from the visible to near-infrared wavelength (520, 940, 1060, and 1310 nm), respectively. The photocurrent varies periodically with the angle of incident polarized light. Fitting with the sine function *I*_ph_(*δ*) = *I*_py_ cos^2^(*δ* + *φ*) + *I*_px_ sin^2^(*δ* + *φ*), the device dichroic ratio *I*_py_/*I*_px_ could be obtained to determine the polarization detection capability of the device; the dichroic ratios of the device under the illumination of 520, 940, 1060, and 1310 nm are 5.53, 6.25, 3.80, and 3.16, respectively. These parameters are higher compared to GeSe photodetectors and other anisotropic devices (shown in Supplementary Table 1)^46,51,52^. The enhancement of the dichroism of GeSe possibly originates from the anisotropic stark effect in GeSe^53^. The GeSe/MoS_2_ Fe-VHJ is a promising polarization-sensitive photodetector for optical sensing.

In conclusion, we have designed a proof-of-concept device architecture, the GeSe/MoS_2_ Fe-VHJ. The carrier transport of the heterojunction can be effectively tuned by the ferroelectric polarization field with band alignment evolution. The device demonstrates quick and sensitive responsivity to visible incident light. Besides, the Fe-VHJ is sensitive in the near-infrared region as well because of the band engineering of ferroelectric dipoles. Ultimately, we used this single device to obtain the intensity and polarization information of light. Our high-performance Fe-VHJ is a pioneering integrated sensing device.

## Methods

### Device fabrication

MoS_2_ and GeSe are exfoliated from bulk materials and transferred on a p-doped silicon substrate covered with 285 nm silicon oxide. Then the chosen GeSe flake was picked up by water-soluble poly(vinyl alcohol) film. The electrodes were defined by electron beam lithography and Ti/Au (15 nm/50 nm) were deposited by thermal evaporation. The P(VDF-TrFE) (70:30 in mol%) was spin-coated on the substrate and annealed at 135 °C for 4 h. The top gate electrode is patterned by a metal mask and 9 nm aluminum was deposited. The top gate is nearly transparent to avoid affecting the light absorption efficiency as shown in Supplementary Fig. 14.

### Electrical and photoelectronic characterization

The electrical measurements were performed by Agilent B2912A and Keysight B1500A at room temperature. The photoresponse measurement was under monochromatic light sources with a spot diameter of about 100 µm to ensure a uniform power on the device. The photocurrent mapping was carried out by a modulated scanning laser beam and the photocurrent signal was acquired by a lock-in amplifier. The response time was measured by a high-speed Tektronix MDO3014 oscilloscope.

## Supplementary information

Supplementary Information

## Data Availability

The authors declare that the data that support the graphs within this paper are available from the corresponding author upon reasonable request. Source data are provided with this paper.

## Source data

Source Data
