# Supplementary material for: Ferroelectric-tuned van der Waals heterojunction with band alignment evolution
Source: Nat Commun. 2021 Jun 29;12:4030. doi: 10.1038/s41467-021-24296-1 (PMC8242043; doi:10.1038/s41467-021-24296-1)
Supplement: Supplementary file 1 — Supplementary Information [file 41467_2021_24296_MOESM1_ESM.pdf]

Supplementary Information

Ferroelectric-tuned van der Waals heterojunction with band alignment evolution

Yan Chen[1,2#], Xudong Wang[1#], Le Huang[3], Xiaoting Wang[3], Wei Jiang[1], Zhen Wang[1], Peng Wang[1], Binmin Wu[1], Tie Lin[1], Hong Shen[1], Zhongming Wei[3*], Weida Hu[1,4*], Xiangjian Meng[1], Junhao Chu[1,2], Jianlu Wang[1,4*]

[1]*State Key Laboratory of Infrared Physics, Shanghai Institute of Technical Physics, Chinese Academy of Sciences, 500 Yu Tian Road, 0.0Shanghai 200083, China.*

[2]*School of Physics and Electronic Science, East China Normal University, Shanghai 200241, China.*

[3]*State Key Laboratory of Superlattices and Microstructures, Institute of Semiconductors, Chinese Academy of Sciences, Beijing 100083, China*

[4]*Hangzhou Institute for Advanced Study, University of Chinese Academy of Sciences, Chinese Academy of Sciences, Hangzhou 310024, China*

[*]Corresponding authors:

Jianlu Wang (email: jlwang@mail.sitp.ac.cn)

Weida Hu (email: wdhu@mail.sitp.ac.cn)

Zhongming Wei (email: zmwei@semi.ac.cn)

[#]These authors contributed equally: Yan Chen, Xudong Wang.

Table of contents

Supplementary Figure 1. Schematic diagram of GeSe structure.

Supplementary Figure 2. Electrical properties of MoS_2 and GeSe FeFET.

Supplementary Figure 3. Effect of P(VDF-TrFE) on the hysteresis in transfer curves of GeSe FET.

Supplementary Figure 4. Evolution of electronic properties of GeSe tuned by P(VDF-TrFE).

Supplementary Figure 5. Photoresponse characterization of GeSe FeFET.

Supplementary Figure 6. Layer-dependent electronic structures of GeSe.

Supplementary Figure 7. Effect of external electric field on the band structure of bulk GeSe calculated by DFT with GGA-PBE functional.

Supplementary Figure 8. Effect of external electric field on the band structure of bulk GeSe calculated by DFT with HSE06 functional.

Supplementary Figure 9. Carrier transport at the interface of GeSe/MoS_2 heterojunction at different polarization states.

Supplementary Figure 10. The effect of the amorphous layer on carrier transport in heterojunction.

Supplementary Figure 11. The noise current spectrum of GeSe/MoS_2 heterojunction.

Supplementary Figure 12. Analysis of photoresponse mechanisms at different wavelengths.

Supplementary Figure 13. Additional photoresponse performance in NIR.

Supplementary Figure 14. Optical image of device structure with transparent top-gate.

Supplementary Table 1. Comparisons of polarization-sensitive photodetectors with related materials and structures.

Supplementary Note 1. Calculation of bandgap evolution of GeSe under an electric field.

Supplementary Note 2. Evolution of electronic properties of GeSe tuned by P(VDF-TrFE).

Supplementary Note 3. Analysis of band alignment of GeSe/MoS_2 Fe-VHJ.

Supplementary Note 4. Thickness effect of GeSe on the tunability of ferroelectric field.

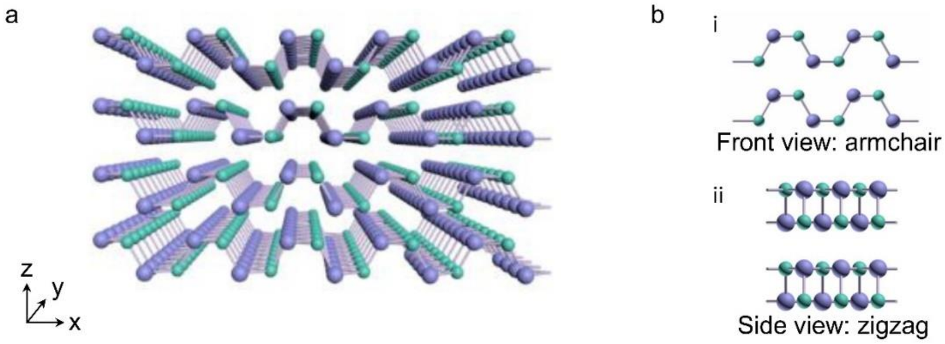

Supplementary Figure 1. Schematic diagram of GeSe structure. (a) Atomic structure of few-layer GeSe. The lattice shows an in-plane wrinkle along the x-axis, resulting in anisotropic structure and properties. (b) Front view and side view of GeSe.

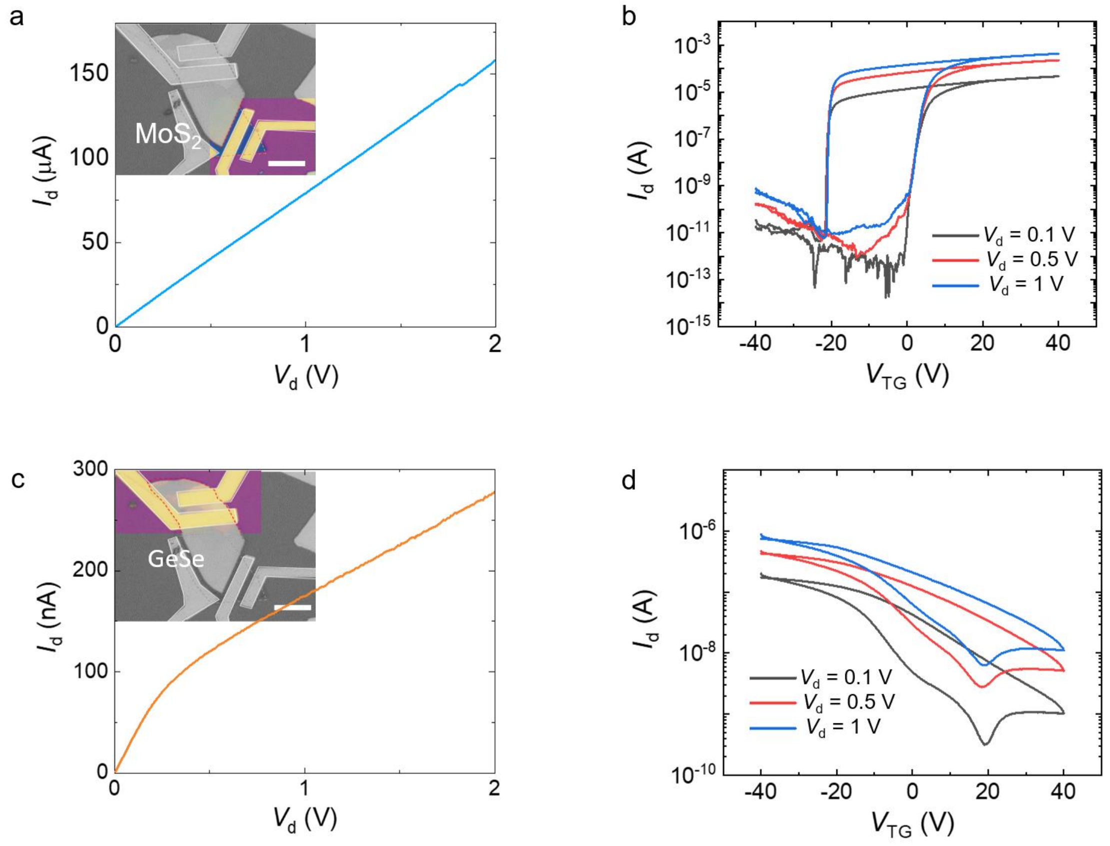

Supplementary Figure 2. Electrical properties of MoS_2 and GeSe FeFET. (a) The output characteristic curve of MoS_2 measured when P(VDF-TrFE) is not polarized. (b) The transfer characteristic curves of MoS_2 FeFET with P(VDF-TrFE) top gate. (c) The output characteristic curve of GeSe measured when P(VDF-TrFE) is not polarized. (d) The transfer characteristic curves of GeSe FeFET tuned by P(VDF-TrFE) top gate.

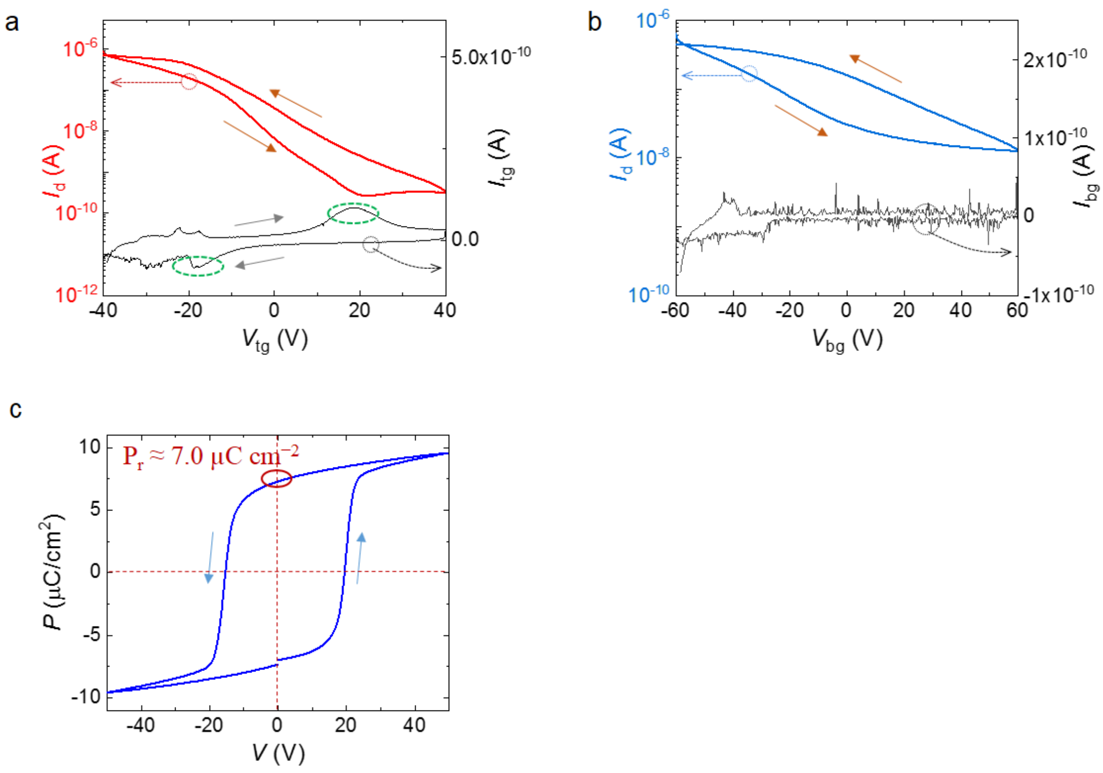

Supplementary Figure 3. Effect of P(VDF-TrFE) on the hysteresis in transfer curves of GeSe FET. Transfer characteristics of GeSe FET tuned by (a) P(VDF-TrFE) top gate and (b) SiO_2 back gate, V_d = 1 V. (c) Ferroelectric hysteresis loop of 200 nm-thick P(VDF-TrFE) film. The coercive voltage is approximately ±20 V.

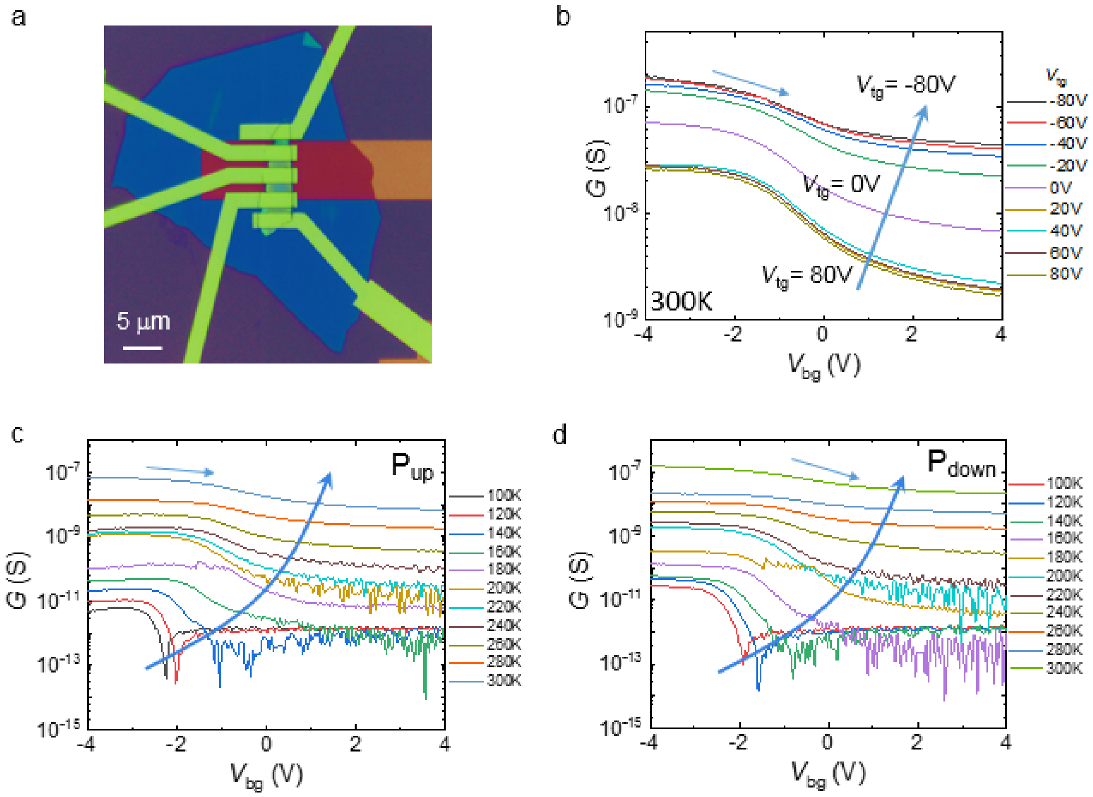

Supplementary Figure 4. Evolution of electronic properties of GeSe tuned by P(VDF-TrFE). (a) The optical image of double-gated GeSe FET with a BN back gate and P(VDF-TrFE) top gate. (b) The conductance as a function of top gate voltage. The temperature-dependency of conductance vs. back gate voltage at (c) P_{up} state and (d) P_{down} state.

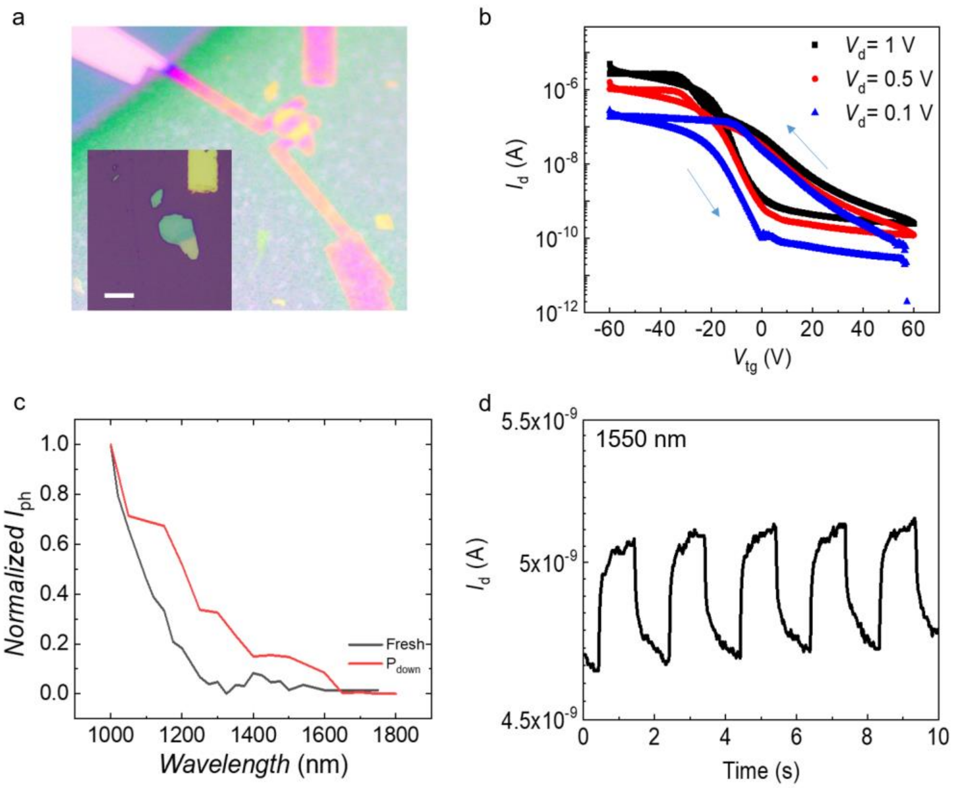

Supplementary Figure 5. Photoresponse characterization of a GeSe FeFET. (a) The optical micrograph of a completed device and the inset is the optical image of the GeSe nanoflake. Scale bar, 10 μm. (b) Transfer characteristics of GeSe FeFET at V_d = 0.1 V, 0.5 V, and 1 V. (c) Photoresponse spectrum of GeSe at Fresh state and P_{down} state. (d) Time-resolved photoresponse with illuminations of 1550 nm.

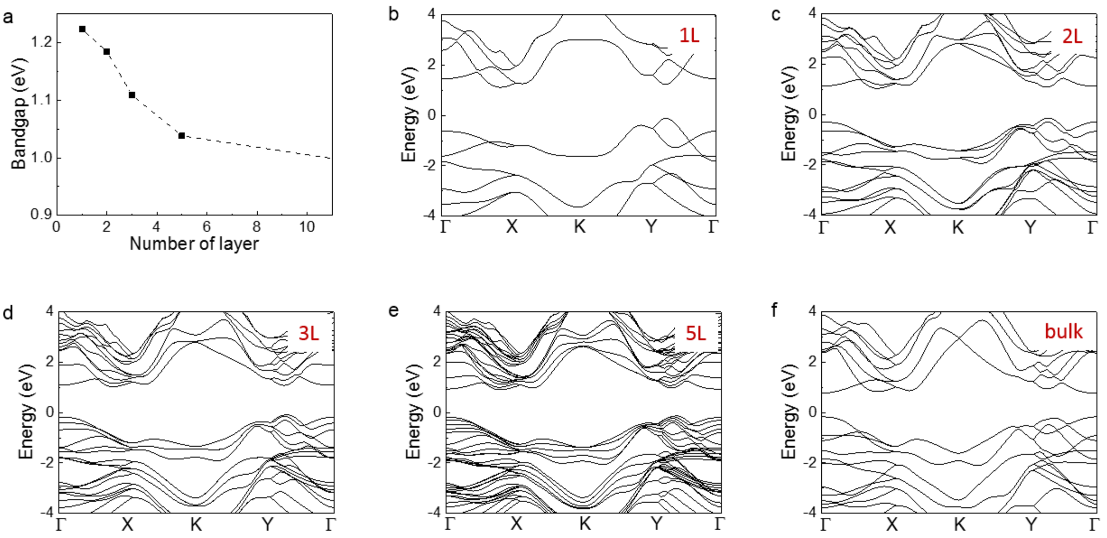

Supplementary Figure 6. Layer-dependent band structures of GeSe. (a) Bandgap as a function of the number of layers. (b) - (f) Electronic band structures of GeSe with different thicknesses calculated by DFT with PBE functional. The bandgap of bulk GeSe is approximately 0.9 eV.

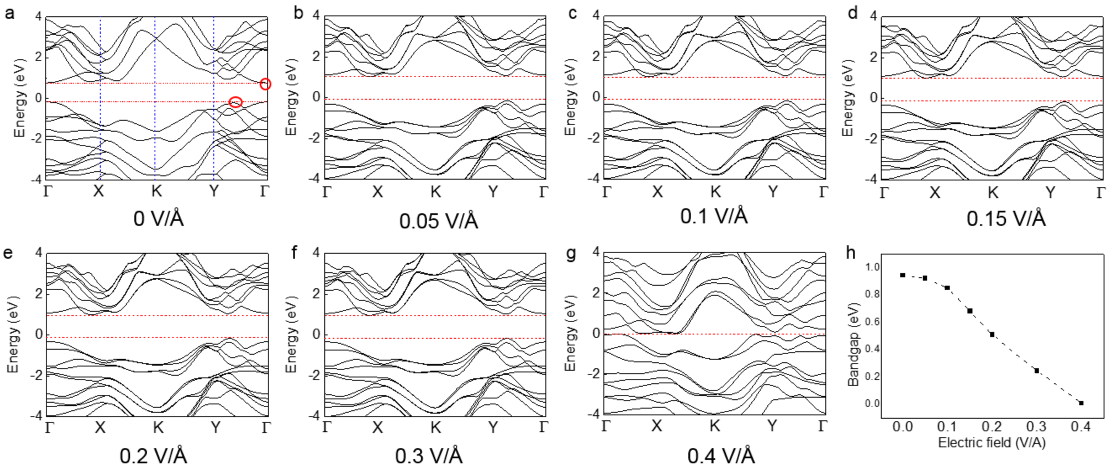

Supplementary Figure 7. **Effect of external electric field on the band structure of bulk GeSe calculated by DFT with PBE functional.** (a)-(g) Calculated band structures of bulk GeSe under external electric field. (h) The electronic bandgap reduces linearly with respect to the external electric field. The slope is approximately 2.2 eVÅV^{-1}.

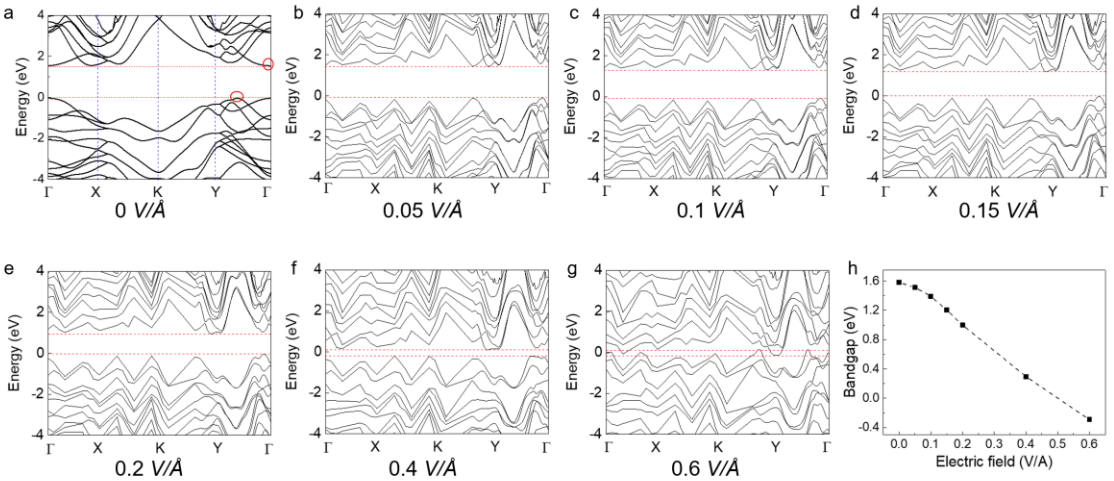

Supplementary Figure 8. Effect of external electric field on the band structure of bulk GeSe calculated by DFT with HSE06 functional. (a)-(g) Calculated band structures of bulk GeSe under external electric field. (h) Electronic bandgap as a function of the applied electric field. The slope is 3.3 eVÅV^{-1} approximately.

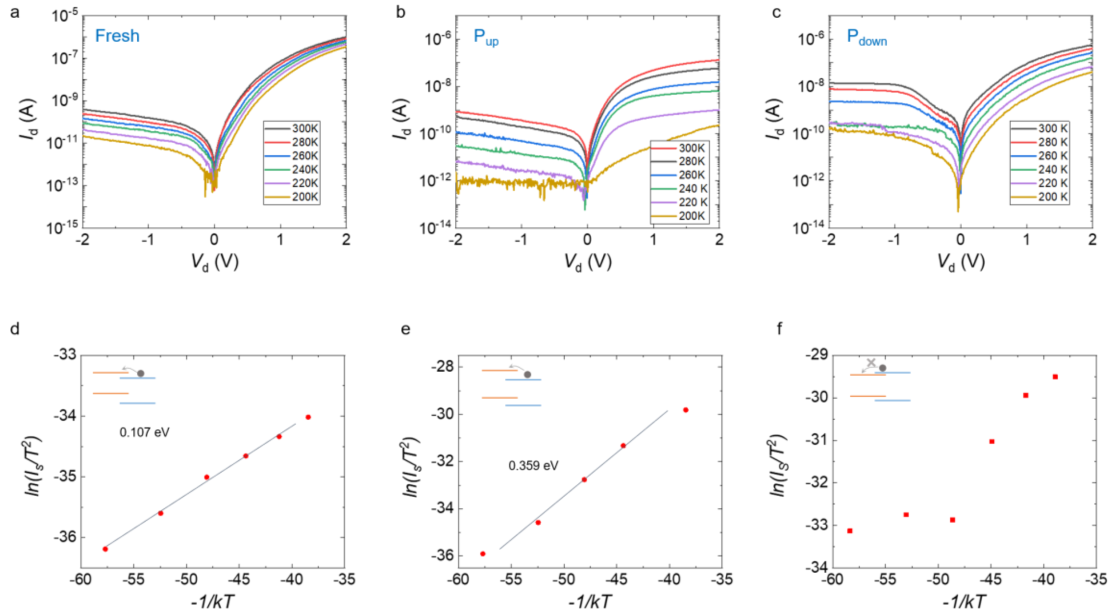

Supplementary Figure 9. **Carrier transport at the interface of GeSe/MoS$_2$ heterojunction at different polarization states.** Temperature-dependent output characteristics at (a) Fresh state, (b) P_{up} state, and (c) P_{down} state. (d)-(f) The thermionic emission model is used to extract band offset at different states. The insets depict the simplified band structure at the interface.

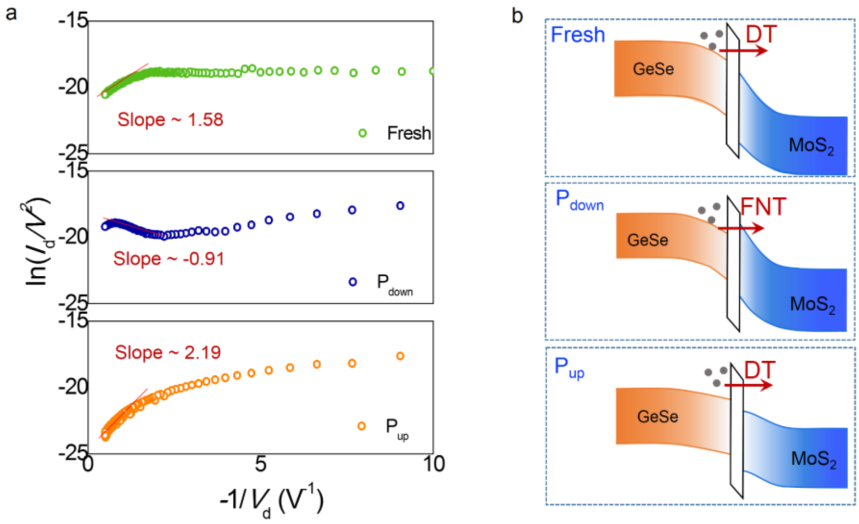

Supplementary Figure 10. The effect of the amorphous layer on carrier transport in heterojunction. (a) $ln(I_d/V^2)$ vs. $-1/V$ curve in the reverse bias regime at different polarization states. (b) Corresponding band diagrams of the heterojunction under different polarization states. The depletion region and the organic residue layer are simplified as a barrier. At fresh state and P_{up} state, the reverse current is dominated by direct tunneling (DT). At P_{down} state, Fowler-Northeim tunneling (FNT) dominates.

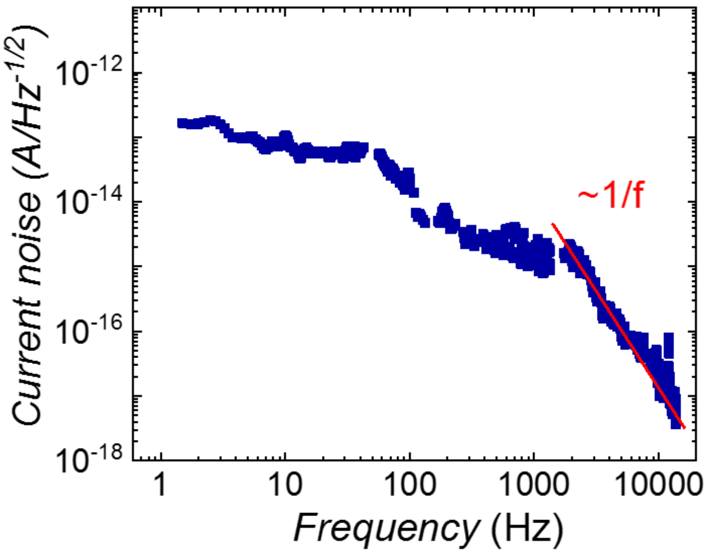

Supplementary Figure 11. The noise current spectrum of GeSe/MoS_2 heterojunction measured with zero bias at room temperature.

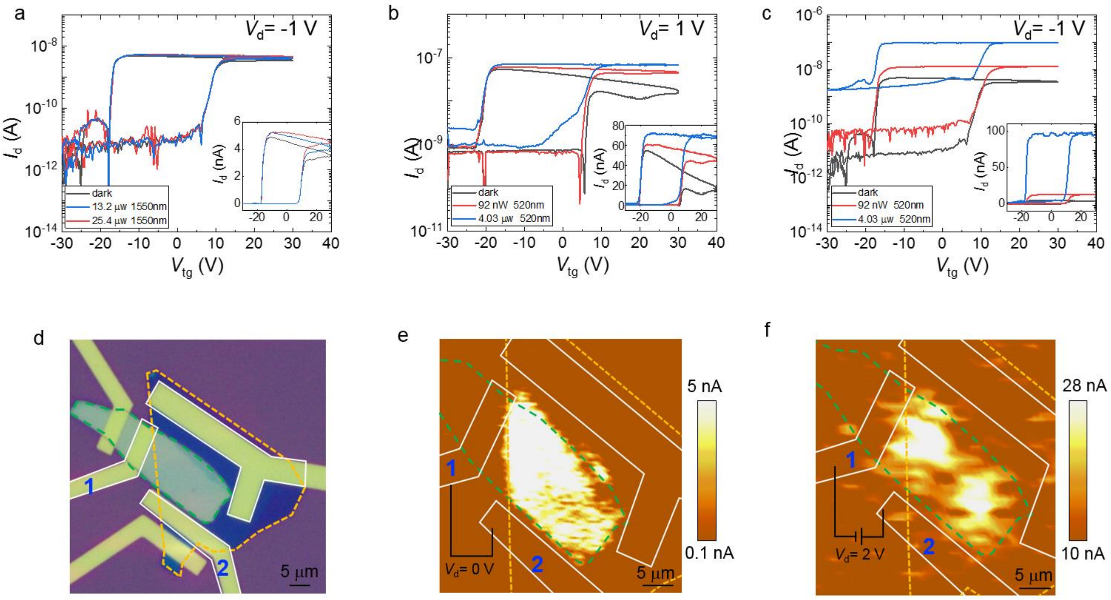

Supplementary Figure 12. Analysis of photoresponse mechanisms at different wavelengths. (a)Transfer characteristics at a reverse bias (V_d = -1 V) with an illumination wavelength of 1550 nm. Transfer characteristics at (b) forward bias (V_d = 1V) and (c) reverse bias (V_d = -1V) under 520 nm laser illumination. The insets show them in linear coordinates. (d) The optical image of a device used to perform photocurrent mapping. Photocurrent mapping images scanning by (e) 520 nm light and (f) 1310 nm light.

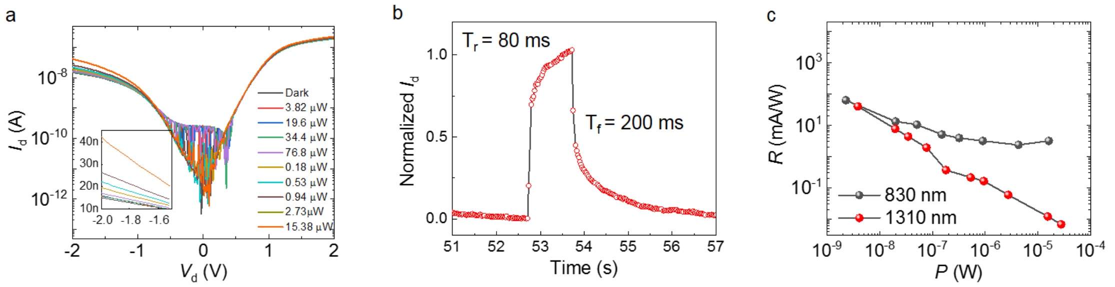

Supplementary Figure 13. Additional photoresponse performance in NIR. (a) output characteristics under the illumination of 1550 nm with different power. (b) Response time measured with illumination of 1550 nm. The rise time is 80 ms and the fall time is approximately 200 ms. (c) Responsivities as a function of incident light power. Excitation is performed with 830 nm and 1310 nm laser diodes.

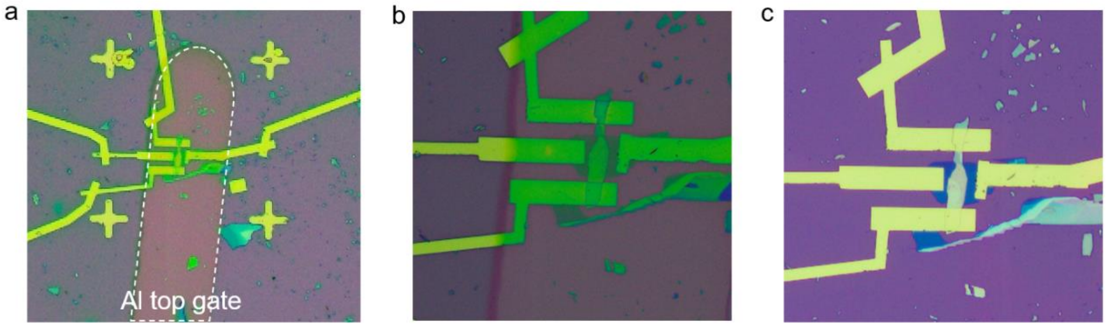

Supplementary Figure 14. Optical image of a device structure with transparent top-gate.

Supplementary Table 1. Comparisons of polarization-sensitive photodetectors with related materials and structures.[1-8]

Device Structure	Working mechanism	R (mA/W)	D* (Jones)	Dichroism ratio	λ (nm)	τ (μs)	Ref.
$GeSe/MoS_2$ Fe-VHJ	PC/PV	729.3	4.7×10^{12}	5.53 (520 nm)<br>6.25 (940 nm)<br>3.80 (1060nm)<br>3.16 (1310nm)	520 - 1550	14	This work
GeSe	PC	4.25	NA	1.09 (520 nm)<br>1.44 (638 nm)<br>2.16 (808 nm)	400 - 950	NA	1
GeAs	PC	NA	NA	1.49 (520 nm)<br>1.14 (623nm)<br>4.4 (830 nm)	400 - 1370	NA	2
$GeSe_2$	PC	NA	NA	3.4 (450 nm)	450 - 530	NA	3
ReS_2	PC	10^3	NA	NA	520	NA	4
BP-on-WSe_2	PC	10^3 (637nm)<br>0.5 (1550nm)	$\sim10^{10}$	5.8 (1550 nm)	400 - 1550	800/800	5
bP	PV	0.35	NA	3.5 (1200 nm)	400 - 3750	40	6
bP/MoS_2	PV	0.9	1.1×10^{10}	~30 (3.5 μm)	2500 - 3800	3.7/4	7
$PdSe_2$/perovskite	PV	0.313	NA	6.04 (808 nm)	200 -1200	3.5/4	8

*PV represents photovoltaic effect and PC represents photoconductive effect.

Supplementary Note 1: Calculation on bandgap evolution of GeSe under an electric field.

The bandgap modulation effect of P(VDF-TrFE) on MoS_2 has been demonstrated in our previous work.[11,12] We can verify the bandgap reduction by measure the cutoff wavelength of the photoresponse. The bandgap of bulk GeSe is approximately 1.1−1.2 eV, which makes its intrinsic response with the cutoff wavelength of 1100 nm. With the modulation of P(VDF-TrFE), however, the detection range of GeSe is broadened to 1550 nm as shown in **Supplementary Fig. 5**.

To estimate the magnitude of bandgap reduction by the Stark effect, we calculate the electronic structure of bulk GeSe under different external electrical fields. First-principle calculations are performed in VASP code within the projector-augmented plane-wave method.[13-15] The general gradient approximation (GGA) of Perdew, Burke, and Ernzerhof (PBE) is adopted to describe the exchange-correlation potential.[16] To overcome the problem of bandgap underestimation in PBE functionals, the HSE06 hybrid functional[17-19] is used to calculate the band structures of layered GeSe. Energy cutoff of 450 eV is employed for the plane-wave basis. In calculations of few-layer GeSe, a vacuum larger than 15 Å is used to eliminate the interaction between adjacent images. The first Brillouin zone is sampled with an $(8 \times 8 \times 1)$ Monkhorst-Pack grid for relaxation of layered GeSe.[20] All the structures are fully relaxed with a force tolerance of 0.01 eV/Å.

From the electronic structures calculated by PBE method as shown in **Supplementary Fig. 6**, the bandgap of GeSe shows a strong dependence on its thickness, which is attributed to quantum confinement effect. With increasing thickness of GeSe, quantum confinement effect becomes less significant, resulting in decreasing bandgap of GeSe. The bandgap of GeSe is calculated to be 0.94 eV, which is close to the reported results.[10] HSE06 calculations obtain larger bandgap of 1.6 eV.

Supplementary Fig. 7 reveals PBE calculation results that bandgap of bulk GeSe exhibits rigid modulation by the external electric field. Following the giant Stark effect, a nearly linear decreasing trend is observed in the bandgap of bulk GeSe. According to the linear dependency of bandgap reduction, the bandgap of bulk GeSe shrinks 0.3 eV

with an external electric field of approximately 0.13 V/Å. The PBE functional calculations were also performed. The result shows that an external field of 0.1 V/Å can make a 0.3 eV reduction of the GeSe bandgap. From the electronic structures of bulk GeSe, it is found that general features of band structures are well preserved when on the GGA-PBE and HSE06 levels. The results given by the two calculation methods are similar and reliable.

The electrostatic field derived from the ferroelectric polarization is estimated by $\sigma = \varepsilon\varepsilon_0 E$, where σ is the charge density at the surface of P(VDF-TrFE) film, which is related to the remnant polarization (P_r) of the ferroelectric materials, ε is the dielectric constant of material (the dielectric constant of GeSe is 6.3), ε_0 is the vacuum permittivity, E is the electric field strength, and P_r is approximately 7.0 μC/cm^2 extracted from the ferroelectric hysteresis loop as shown in **Supplementary Fig. 3c**. The remnant polarization electric field intensity is calculated to be 1.26×10^9 V/m, which may induce a 0.3 eV bandgap reduction in GeSe according to the calculated value.

Supplementary Note 2: The evolution of electronic properties of GeSe tuned by P(VDF-TrFE).

We explored the evolution in the electronic properties of GeSe with the applied top gate voltage (V_{tg}). Conductance measurements were performed in dual-gated GeSe transistors with a BN back gate and P(VDF-TrFE) top gate as shown in **Supplementary Fig. 8**. The tendency of the minimal conductance moves in the same direction as the top gate voltage. When a negative top gate voltage is applied ($V_{tg} < 0$ V, holes accumulate in the GeSe), the conductance increases with additional holes filling the valence states. As a result of the holes accumulation, the gate electric field is screened and the bandgap of GeSe remains approximately constant. On the other hand, a positive top gate voltage injects electrons into the GeSe flake and the conductance reduces. The depleted GeSe facilitates the ferroelectric field to penetrate the whole GeSe flake, causing the bandgap reduction due to the giant Stark effect.[21]

The impact of the remnant polarization field on the GeSe was also investigated. The density of thermally generated intrinsic carriers (n_i) reveals the bandgap of a semiconductor. Their relationship is given by $n_i \propto \exp(-E_g/2kT)$, where E_g is the bandgap of GeSe and k is the Boltzmann constant. Meanwhile, the intrinsic carrier density can be obtained by the minimal conductance at the charge neutrality state.[22] They follow the equation of $\sigma_m = qn_i\mu_h$. The temperature-dependency of conductance at P_{up} and P_{down} states are plotted in **Supplementary Fig. 8c and 8d**. At high temperatures, it is difficult to find the minimum conductance, but the data shows an increasing trend of conductance with increasing temperature. More importantly, the conductance at P_{down} state is always larger than that of P_{up} state. Consequently, the density of intrinsic carriers at P_{down} state is higher, indicating a smaller E_g of GeSe.

Supplementary Note 3: **Analysis of band alignment of GeSe/MoS_2 VHJ.**

1. To better understand the transport properties of the GeSe/MoS_2 heterojunction, we analyzed the electrical properties of MoS_2 and GeSe, respectively. To determine its band alignment, we calculated the mobility, carrier concentration, and the Fermi level positions of these two materials based on the measured data as shown in **Supplementary Fig. 2**. The specific methods are as follows.

2. The mobility of MoS_2 and GeSe were calculated by $\mu = (L/(W\cdot\varepsilon_0\varepsilon_r/d\cdot V_d)) \times dI/dV_{tg}$, where L and W are the channel length and width, ε_r and d are the dielectric constant and thickness of P(VDF-TrFE), respectively. μ_{MoS2} = 92.16 $cm^2V^{-1}s^{-1}$, μ_{GeSe} = 0.135 $cm^2V^{-1}s^{-1}$.

The carrier concentration was calculated by $n(p) = \sigma/\mu e$, where σ is the conductivity derived from I_d-V_d curves, and e is the elementary electronic charge. The concentrations of MoS_2 (electrons) and GeSe (holes) were 3.21×10^{10} cm^{-2} and 1.63×10^{12} cm^{-2}, respectively.

3. The carrier concentration of conduction band and valence band can be also calculated as follows[9]:

$$n = (g_{2D}k_BT)ln\{1+exp[(E_F-E_C)/ k_BT]\}, \quad (1)$$

$$p = (g_{2D}k_BT)ln\{1+exp[-(E_F-E_V)/ k_BT]\}, \quad (2)$$

where g_{2D} is the density of states, k_B is the Boltzmann constant, T is the temperature, E_F-E_C and E_F-E_V are the separations from Fermi level to conduction band minima (CBM) and valence band maxima (VBM), which we need to obtain by calculation.

4. The density of states can be estimated by formula:

$$g_{2D} = 4\pi m_{e/h}^*/h^2 \quad (3)$$

according to references, $m_{e,\ MoS2}^* = 0.48m_0$, $m_{h,\ GeSe}^* = 0.75m_0$, the Plank constant $h = 6.626 \times 10^{-34}$ J·s,

$g_{2D,MoS2} = 4 \times 3.14 \times 0.48 \times 9.11 \times 10^{-31} /(6.626 \times 10^{-34})^2 = 1.25 \times 10^{37}$,

$g_{2D,GeSe} = 4 \times 3.14 \times 0.75 \times 9.11 \times 10^{-31}/(6.626 \times 10^{-34})^2 =1.95 \times 10^{37}$.

The Boltzmann constant $k_B = 1.38 \times 10^{-23}$ J/K, temperature T = 300 K, the calculated carrier concentration (n, p), and density of states g_{2D} are taken into equation (1) and (2). As a result, the E_F-E_v of GeSe is 0.27 eV, E_C-E_F of MoS_2 is 0.38 eV.

The bandgap and the electron affinity energy of GeSe are 1.1 eV and 4.1 eV, respectively. The bandgap and the electron affinity energy of MoS_2 are 1.2 eV and 4.2 eV, respectively. Therefore, the band structure of GeSe/MoS_2 heterojunction at Fresh state can be illustrated as shown in Fig. 2f.

As shown in Supplementary Figure 9, the *I-V* characteristics of the heterojunction are temperature-dependent. Here, we use the thermionic emission model to extract the band offset:[10]

$$I = I_S\left[\exp\left(\frac{eV}{\eta k_B T}\right) - 1\right],$$

$$I_S \propto T^2 \exp\left(-\frac{e\varphi_B}{k_B T}\right),$$

where I_s is the saturation current, k_B is the Boltzmann constant, V is the applied voltage, T is the temperature, η is the ideality factor, and e is the electron charge. φ_B is the barrier that electrons need to overcome as shown in Supplementary Figure 9d-f. It can be extracted from the slope in the plot of $\ln(I_s/T^2)$ *vs* $-1/kT$. The band offset under Fresh state is 0.107 eV, which is consistent with our previous result (0.1 eV). The band offsets at the "P_{up}" state and "P_{down}" state are fitted to be 0.359 eV and -0.051 eV, respectively. It indicates that the polarization of P(VDF-TrFE) effectively tuned the band structure of the GeSe/MoS_2 heterojunction. The variation tendency of the band offset is illustrated in Fig. 2g and Fig. 2h.

Supplementary Note 4: Thickness effect of GeSe on the tunability of ferroelectric field.

The Debye length of the GeSe can be calculated by $L_D = \sqrt{\frac{\varepsilon_s kT}{q^2 N}}$, where the ε_s is the permittivity of GeSe, k is the Boltzmann's constant, q is the charge of the electron, and N is the carrier concentration. $N = N_c \exp\left(-\frac{E_F - E_V}{kT}\right) = \frac{2(2\pi m^* kT)^{3/2}}{h^3} \exp\left(-\frac{E_F - E_V}{kT}\right)$, At fresh state, the E_F-E_V of GeSe is 0.27 eV, the hole concentration is 5.2×10^{15} cm^{-3} and the Debye length is 41.8 nm. Since we have confirmed that the band alignment changed when P(VDF-TrFE) is polarized down, where the GeSe is depleted. So, the carrier density can be calculated as $N = N_c \exp\left(-\frac{E_g}{2kT}\right) = \frac{2(2\pi m^* kT)^{3/2}}{h^3} \exp\left(-\frac{E_g}{2kT}\right)$ and the result is 9.4×10^9 cm^{-3}. As a result, the Debye length of GeSe with downward polarization is calculated to be 30.9 μm. So, it is reasonable to conclude that the downward polarization electric field can penetrate the GeSe hundreds of nanometers thick and modulate the interfacial band alignment.

References:

1. Wang, X. *et al.* Short-wave near-infrared linear dichroism of two-dimensional germanium selenide. *J. Am. Chem. Soc.* **139**, 14976-14982 (2017).
2. Yang, Y. *et al.* Air-stable in-plane anisotropic $GeSe_2$ for highly polarization-sensitive photodetection in short wave region. *J. Am. Chem. Soc.* **140**, 4150-4156 (2018).
3. Zhou, Z. *et al.* Perpendicular optical reversal of the linear dichroism and polarized photodetection in 2D GeAs. *ACS Nano* **12**, 12416-12423 (2018).
4. Liu, F. *et al.* Highly sensitive detection of polarized light using anisotropic 2D ReS_2. *Adv. Func. Mater.* **26**, 1169-1177 (2016).
5. Ye, L. *et al.* Highly polarization sensitive infrared photodetector based on black phosphorus-on-WSe_2 photogate vertical heterostructure. *Nano Energy* **37**, 53-60 (2017).
6. Yuan, H. *et al.* Polarization-sensitive broadband photodetector using a black phosphorus vertical p-n junction. *Nat. Nanotechnol.* **10**, 707-713 (2015).
7. Bullock, J. *et al.* Polarization-resolved black phosphorus/molybdenum disulfide mid-wave infrared photodiodes with high detectivity at room temperature. *Nat. Photon.* **12**, 601-607 (2018).
8. Zeng, L. H. *et al.* Multilayered $PdSe_2$/perovskite Schottky junction for fast, self-powered, polarization-sensitive, broadband photodetectors, and image sensor application. *Adv. Sci.* **6**, 1901134 (2019).
9. Chen, J. W. *et al.* A gate-free monolayer WSe2 pn diode. *Nat. Commun.* **9**, 3143 (2018).
10. Yap, W. C. *et al.* Layered material GeSe and vertical GeSe/MoS_2 p-n heterojunctions. *Nano Res.* **11**, 420-430 (2017).
11. Wang, X. *et al.* Ultrasensitive and broadband MoS_2 photodetector driven by ferroelectrics. *Adv. Mater.* **27**, 6575-6581 (2015).
12. Chen, Y. *et al.* Optoelectronic properties of few-layer MoS_2 FET gated by ferroelectric relaxor polymer. *ACS Appl. Mater. Interfaces* **8**, 32083-32088 (2016).
13. Blochl, P. E. Projector augmented-wave method. *Phys. Rev. B* **50**, 17953-17979

(1994).

14. Kresse, G. *et al.* Efficient iterative schemes for ab initio total-energy calculations using a plane-wave basis set. *Phys. Rev. B* **54**, 11169-11186 (1996).

15. Kresse, G. *et al.* From ultrasoft pseudopotentials to the projector augmented-wave method. *Phys. Rev. B* **59**, 1758-1775 (1999).

16. Perdew, J. P. *et al.* Generalized gradient approximation made simple. *Phys. Rev. Lett.* **77**, 3865-3868 (1996).

17. Heyd, J. *et al.* Hybrid functional based on a screened coulomb potential. *J. Chem. Phys.* **118**, 8207 (2003).

18. Heyd, J. *et al.* Energy band gaps and lattice parameters evaluated with the Heyd-Scuseria-Ernzerhof screened hybrid functional. *J. Chem. Phys.* **123**, 174101 (2005).

19. Heyd, J. *et al.* Hybrid functionals based on a screened Coulomb potential. *J.Chem. Phys.* **118**, 8207-8215 (2003).

20. Chadi, D. J. *et al.* Special points in the Brillouin zone. *J. Phy. Rev.* **8**, 5747-5753 (1976).

21. Liu, Y. *et al.* Gate-tunable giant Stark effect in few-layer black phosphorus. *Nano Lett.* **17**, 1970-1977 (2017).

22. Deng, B. *et al.* Efficient electrical control of thin-film black phosphorus bandgap. *Nat. Commun.* **8**, 14474 (2017).
